# Notch Signaling Ligand Jagged1 Enhances Macrophage-Mediated Response to *Helicobacter pylori*

**DOI:** 10.3389/fmicb.2021.692832

**Published:** 2021-07-08

**Authors:** Junjie Wen, Chuxi Chen, Meiqun Luo, Xiaocong Liu, Jiading Guo, Tingting Wei, Xinyi Gu, Sinan Gu, Yunshan Ning, Yan Li

**Affiliations:** ^1^School of Laboratory Medicine and Biotechnology, Southern Medical University, Guangzhou, China; ^2^The First Clinical Medical School, Southern Medical University, Guangzhou, China

**Keywords:** *Helicobacter pylori*, macrophage, Notch signaling, Jagged1, bactericidal activities

## Abstract

*Helicobacter pylori* (*H. pylori*) is one of the gram-negative bacteria that mainly colonize the stomach mucosa and cause many gastrointestinal diseases, such as gastritis, peptic ulcer, and gastric cancer. Macrophages play a key role in eradicating *H. pylori*. Recent data have shown that Notch signaling could modulate the activation and bactericidal activities of macrophages. However, the role of Notch signaling in macrophages against *H. pylori* remains unclear. In the present study, in the co-culture model of macrophages with *H. pylori*, the inhibition of Notch signaling using γ-secretase decreased the expression of inducible nitric oxide synthase (iNOS) and its product, nitric oxide (NO), and downregulated the secretion of pro-inflammatory cytokine and attenuated phagocytosis and bactericidal activities of macrophages to *H. pylori*. Furthermore, we identified that Jagged1, one of Notch signaling ligands, was both upregulated in mRNA and protein level in activated macrophages induced by *H. pylori*. Clinical specimens showed that the number of Jagged1^+^ macrophages in the stomach mucosa from *H. pylori*-infected patients was significantly higher than that in healthy control. The overexpression of Jagged1 promoted bactericidal activities of macrophages against *H. pylori* and siRNA-Jagged1 presented the opposite effect. Besides, the addition of exogenous rJagged1 facilitated the pro-inflammatory mediators of macrophages against *H. pylori*, but the treatment of anti-Jagged1 neutralizing antibody attenuated it. Taken together, these results suggest that Jagged1 is a promoting molecule for macrophages against *H. pylori*, which will provide insight for exploring Jagged1 as a novel therapeutic target for the control of *H. pylori* infection.

## Introduction

*Helicobacter pylori* (*H. pylori*) is a microaerophilic, gram-negative bacterium that mainly colonizes the gastric mucosa of humans. It is estimated that more than 50% of the human population is infected with *H. pylori*, which leads to various gastrointestinal diseases, including gastritis, peptic ulcer, and gastric cancer (Guevara and Cogdill, 2020). Importantly, long-term infection is a risk factor for the development of gastric adenocarcinoma and *H. pylori* is listed as a Class I carcinogen (Wang et al., 2014; Hooi et al., 2017). *H. pylori* infection often induces an immune response, which seems to be insufficient to completely combat pathogens and even causes lifelong infection. Even though antibiotic-based therapy is still the most effective treatment for controlling infection, it is not feasible for large-scale control mainly because of drug resistance, refractory, and poor compliance in *H. pylor*i-infected patients. Therefore, it is essential to better understand the pathogenesis of *H. pylori* infection and to develop novel therapeutic strategies for its eradication.

*H. pylori* infection is initially recognized by the innate immune system. Macrophages are one of key cells of innate immunity and play an essential role in host immune defense against *H. pylori* infection and in the regulation of inflammatory processes (Kaparakis et al., 2008). *H. pylori* often induce the aggregation and activation of macrophages in the gastric mucosa, forming the first line of immune defense (Krakowiak et al., 2015). An important feature of macrophages is their diversity and plasticity. During *H. pylori* infection, macrophages are typically polarized to M1 and produce the enzyme, inducible nitric oxide synthase (iNOS), which promotes the production of NO and pro-inflammatory cytokines such as IL1β, IL6, and IL12p40 (Wilson et al., 1996; Gobert et al., 2004; Odenbreit et al., 2006; Rad et al., 2007; Fehlings et al., 2012).

The regulatory mechanism of macrophage activation induced by pathogens is highly complex, and many signaling pathways are involved in this process such as TLR, NF-κB, and STAT1 (Xu et al., 2012). Recently, Notch signaling has been considered to regulate the activation of macrophages (Keewan and Naser, 2020b). It is an evolutionary conserved pathway that is involved in cell fate decision, proliferation, and survival (Kopan and Ilagan, 2009; Aster et al., 2017). In general, there are four receptors (Notch1, Notch2, Notch3, and Notch4) and five ligands (DLL1, DLL3, DLL4, Jagged1, and Jagged2) in mammals. Once bound by ligands, the receptors undergo subsequent proteolytic cleavage *via* γ-secretase and release the intracellular domain of Notch (NICD), which is translocated into the nucleus and forms a complex with the transcription mediators of the recombination signal-binding protein for immunoglobulin κJ region (RBP-κJ) to induce transcriptional expression of downstream target genes such as Hes1 (Radtke et al., 2013; Palmer and Deng, 2015). There is accumulating evidence that pathogens induce the expression of Notch receptors and ligands in macrophages with subsequent activation of Notch signaling that contributes to cytokine production (Monsalve et al., 2006, 2009; Hu et al., 2008; Narayana and Balaji, 2008; Palaga et al., 2008; Bansal et al., 2009; Kapoor et al., 2010). Additionally, Notch signaling in macrophages assists in eradicating microbe infections. For example, *Mycobacteria* infection induces macrophage Notch1 upregulation, which is involved in iNOS expression (Narayana and Balaji, 2008; Kapoor et al., 2010). The expression of the Notch1 receptor on macrophages is induced and involves TLR4, resulting in the modulation of the production of IL6 to combat *Paracoccidioides brasiliensis* infection (Romera et al., 2017). DLL4-triggered Notch signaling promotes pro-inflammatory macrophage activation *in vitro* and *in vivo* (Fung et al., 2007; Fukuda et al., 2012; Koga et al., 2015; Nakano et al., 2016, 2019). Although the current findings suggest a possible link between Notch activation and an inflammatory environment in many disease states, studies on the possible interplay between Notch signaling and inflammation in the context of macrophage are limited (Xu et al., 2015). To date, the expression pattern and the role of Notch signaling in macrophages in response to *H. pylori* infection remain unknown.

In the present study, we demonstrated the upregulation of Jagged1 in activated macrophages in an *in vitro* co-culture system of macrophages and *H. pylori*, which resulted in an increase in pro-inflammatory mediators and phagocytosis and a decrease in bacterial load that together imparted anti-bacterial activity in macrophages. The inhibition of the Notch/Jagged1 signal by γ-secretase inhibitor (DAPT) in macrophages decreased pro-inflammatory mediator secretion and phagocytosis and subsequently increased *H. pylori* load. Additionally, the infiltration of Jagged1^+^ CD68^+^ cells was higher in the gastric mucosa of *H. pylori*-positive gastritis patients compared with negative controls (NCs). The overexpression of Jagged1 in macrophages and exogenous rJagged1 facilitated the production of pro-inflammatory mediators and the bactericidal activities of macrophages to *H. pylori*, whereas the opposite was observed with siRNA-Jagged1 knockdown and Jagged1 antibody. Taken together, the results showed for the first time that *H. pylori* induce the expression of Jagged1 in macrophages, which subsequently influences anti-bacterial immunity, suggesting that Jagged1 may be a novel target for modifying the immune response against *H. pylori* infection.

## Materials and Methods

### *Helicobacter pylori* Culture

The *H. pylori* strain SS1 cells were cultured on *H. pylori*-based blood agar plates supplemented with 7% sterile defibrillated sheep blood, 5 μg/ml trimethoprim, 800 ng/ml amphotericin B, 400 μg/ml vancomycin hydrochloride, 200 μg/ml cefsulodin sodium, and 17.5 ng/ml polymyxin B sulfate at 37°C under microaerobic conditions (5% O_2_, 10% CO_2_, and 85% N_2_). The cells were collected and washed twice with sterilized PBS, and then resuspended with PBS to determine the density of *H. pylori* by measuring the optical density at a wavelength of 600 nm [1 OD_600_ = 1 × 10^9^ colony-forming units (CFU)/ml].

### Cell Culture

The murine macrophage cell line RAW264.7 (American Type Culture Collection,TIB-71) was maintained in Dulbecco’s modified Eagle’s medium (DMEM) supplemented with 10% fetal bovine serum (FBS), 100 U/ml penicillin, and 100 μg/ml streptomycin in humidified 5% CO_2_ at 37°C. About 4 × 10^5^ cells were incubated in a 12-well plate overnight before experimentation. THP-1, human monocytic leukemia cell line (American Type Culture Collection, TIB202), was maintained in RPMI-1640 supplemented with 10% FBS, 100 U/ml penicillin, and 100 μg/ml streptomycin in humidified 5% CO_2_ at 37°C and was incubated with 320 nM phorbol 12-myristate 13-acetate (PMA) for 24 h to allow differentiation into human macrophage-like cells. Then, the cells were washed with PBS twice and incubated overnight before experimentation.

### Generation of Human Monocyte-Derived Macrophages (HMDM)

The peripheral blood mononuclear cells (PBMCs) were isolated from healthy donors by Ficoll density-gradient centrifugation (TBD Sciences, Shanghai, China). Monocytes were purified from PBMCs using anti-CD14 microbeads (BD PharMingen, San Diego, CA, United Sstates), according to the manufacturer’s instructions and cultured in RPMI-1640 medium (Invitrogen, Carlsbad, CA, United States) with 10% human type AB serum. Then, the CD14^+^ monocytes were differentiated into macrophages by incubating in RPMI-1640 medium (Invitrogen) with 10% human AB serum. After 7 days, the macrophages were harvested and used in the subsequent experiments.

### Co-culture Model of Macrophages With *Helicobacter pylori*

The macrophages (4 × 10^5^ cells/well for RAW264.7, 4 × 10^5^ cells/well for THP1, and 2 × 10^5^ cells/well for HMDM) were incubated in fresh medium supplemented with 10% FBS at 37°C with 5% CO_2_. For all infection experiments, *H. pylori* was directly added to the RAW264.7 or THP1 cell culture at a multiplicity of infection (MOI) of 50 CFU/cell and HMDM at a MOI of 10 CFU/cell. For the uninfected groups, the equivalent volume of PBS was added into the control cells.

### RNA Extraction and qPCR Analysis

Total RNA was isolated from cells using Raito Plus method (TAKARA, Shiga, Japan). cDNA was prepared from each sample using 1 μg of RNA and a PrimeScript RT reagent Kit (Vazyme, Nanjing, China) according to the manufacturer’s instructions. The real-time polymerase chain reaction (PCR) was performed for 40 cycles to amplify target genes in triplicate using PrimeScript II Reverse Transcriptase (Vazyme) and detected target genes using a Lightcycler96 (Roche, Mannheim, Germany) following the manufacturer’s instructions. To assess gene expression, the relative quantitation of target gene expression was determined by the ΔΔCt (threshold cycle) method. ΔCt is the difference between the Ct of the target mRNA and that of the internal control for each group. The specific primers for target genes used in this study are listed in Supplementary Table 1.

### Protein Extraction and Western Blotting

Total proteins from macrophages were extracted using RIPA buffer (Genstar, Chino, CA, United States) supplemented with 10 nM PMSF (Genstar). The protein samples were resuspended in 5 × SDS loading buffer (Genstar) and incubated in a boiling water bath for 10 min. After sodium dodecyl sulfate–polyacrylamide gel electrophoresis (SDS-PAGE) separation, the proteins were transferred onto polyvinylidene difluoride membranes (Merck, Darmstadt, Germany). The primary and secondary antibodies used in this study are listed in Supplementary Table 2. The densitometry was performed by Gel-Pro Analyzer, version 4.0 (Media Cybernetics, Rockville, MD, United States).

### CFU Assay

To determine the bactericidal activities of macrophages, *H. pylori* was collected from the supernatant of the co-culture model and plated it on a sheep blood agar plate at 37°C under microaerophilic conditions for 7 days. Then, the number of CFU of *H. pylori* was counted.

### Texas Red Staining of *Helicobacter pylori*

To generate the Texas Red-labeled *H. pylori*, *H. pylori* was grown in Brucella broth with 5% FBS and 0.01% Texas Red (Sigma, St. Louis, MO, United States) with gentle shaking at 37°C under microaerobic conditions for 16 h. Then, the Texas Red-labeled *H. pylori* was collected and added directly to the cell culture at an MOI of 50 and incubated for 3 h at 37°C with 5% CO_2_.

### Examination of Phagocytosis Ability

To determine phagocytosis ability, about 4 × 10^5^ RAW264.7 cells were incubated in a 12-well plate with fresh DMEM medium supplemented 10% FBS at 37°C with 5% CO_2_. Then, the RAW264.7 cells were co-cultured with Texas Red-labeled *H. pylori* at an MOI of 50. The intensity of the dye was measured using a fluorescent microscope. For flow cytometry, the macrophages were incubated as previously described for Texas Red-labeled *H. pylori*. All the macrophages were collected and washed in PBS. The fluorescence intensity of Texas Red was measured by using a flow cytometer (BD LSRFortessa X-20, United States), and the mean fluorescence intensity (MFI) was analyzed with FlowJo software (BD PharMingen).

### Enzyme-Linked Immunosorbent Assay of Cytokines

The concentration of murine and human cytokines IL-1β, IL6, IL-10, IL-12p40, IFNγ, TNFα, and TGFβ was measured using an enzyme-linked immunosorbent assay (ELISA) kit (Bioswamp, Wuhan, China) according to manufacturer’s instructions.

### Measurement of NO Production

The NO production was assessed according to the level of nitrite formed in the supernatant of cells by using Griess reagent (Beyotime, Beijing, China). The nitrite concentrations were calculated from a standard curve derived from the reaction of NaNO_2_ in the assay.

### Immunofluorescence Staining of CD68 and Jagged1

The protocol conformed with the Institutional Human Ethics Review Board of Nanfang Hospital, Southern Medical University. Human stomach tissue biopsies were obtained from the Department of Gastroenterology. The immune-fluorescence staining for CD68 and Jagged1 in the gastric mucosa of *H. pylori*-uninfected (Six cases) and *H. pylori*-infected gastritis patients (10 cases) was performed by using a standard staining protocol. The tissues were blocked with 5% FBS for 30 min at room temperature, followed by successive incubation with primary and secondary antibodies for CD68 and Jagged1. The secondary goat anti-rabbit antibody conjugated with fluorescein isothiocyanate (FITC) and goat anti-mouse antibody conjugated with Cy3 were used. Slides were imaged on a microscope (E800, Nikon, Tokyo, Japan). The CD68^+^ Jagged1^+^ cells were captured and counted. Images were all modestly adjusted in terms of brightness and contrast in Adobe Photoshop CC 2017 (Adobe, San Jose, CA, United States).

### Treatment for Macrophages

We firstly determined the optimum concentration of DAPT (S2215, Selleck, Shanghai, China) to inhibit Notch signaling. Briefly, RAW264.7 cells were treated with DAPT (0, 10, 20, and 40 μM) for 24 h. The mRNA expression of Hes1, the downstream gene of Notch signaling, was evaluated by qPCR. The result showed that DAPT inhibited the expression of Hes1 in a dose-dependent manner and 40 μM was maximum inhibition efficiency (Supplementary Figure 1). Therefore, we used this concentration in the following experiments. For inhibition of Notch signaling, macrophages were pretreated with 40 μM DAPT for 24 h or DMSO as control. For overexpression of Jagged1 in macrophages, macrophages were transfected with 1000 ng of plasmid pCMV-tag4-Jagged1 or NC plasmid pCMV-tag4 and mixed with 2 μl of Lipofectamine 3000 Transfection Reagent (Invitrogen, Carlsbad, CA, United States) for 48 h according to the manufacturer’s instructions. For downregulation of Jagged1 in macrophages, macrophages were transfected with 200 μM Jagged1-specific or NC siRNAs and mixed with 2 μl of Lipofectamine 3000 Transfection Reagent for 24 h, according to the manufacturer’s instructions. For the addition of rJagged1, RAW267.4 cells were treated with recombinant mouse Jagged1 protein (rJagged1, 10 ng/ml) or normal immunoglobulin G (IgG) control for 24 h. For neutralization studies, RAW264.7 cells were cultured in 12-well plates in 3 μg/ml Jagged1 neutralizing antibody or normal IgG control for 1 h.

### Statistical Analysis

The statistical analysis was performed by using GraphPad Prism 6.0 software (GraphPad Software). The data are presented as the mean ± SD. At least three biological replicates were performed for all studies using cell culture. The statistical differences between mean values were evaluated by using Student’s *t*-test. The results were considered statistically significant with *p* (^∗^*p* < 0.05, ^∗∗^*p* < 0.01 and ^∗∗∗^*p* < 0.001).

## Results

### Inhibition of Notch Signaling in Macrophages Attenuates the Production of iNOS and Pro-inflammatory Cytokines

**FIGURE 1:**
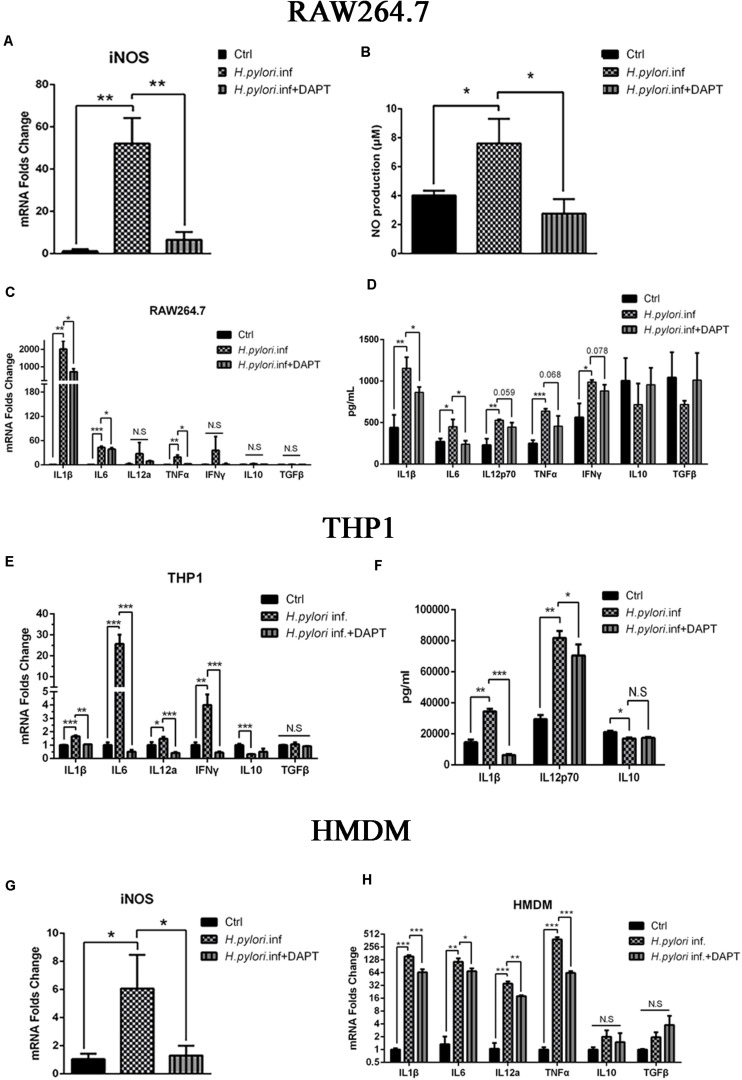
Inhibition of Notch decreases the expression of pro-inflammatory mediators in macrophages during *Helicobacter pylori (H. pylori*) infection. Macrophages were pretreated with DAPT for 12 h and then co-cultured with *H. pylori* (MOI = 50) for 12 h. DMSO (0.08%) was used as control for DAPT. PBS was used as control for *H. pylori* infection. mRNA was extracted from macrophages. **(A)** qPCR was performed to examine mRNA expression of iNOS in RAW264.7. **(B)** Griess was performed to assess the production of NO in the supernatant of the co-culture. **(C)** qPCR was performed to examine the mRNA expression of proinflammatory cytokines IL-1β, IL-6, IL12a, TNF-α, and IFNγ, and anti-inflammatory cytokines IL-10 and TGF-β in RAW264.7. **(D)** ELISA was performed to examine the protein levels of proinflammatory cytokines IL-1β, IL-6, IL-12p70, TNF-α, and IFN-γ and anti-inflammatory cytokines IL-10 and TGF-β in the supernatant of the *H. pylori*-RAW264.7 co-culture. **(E)** qPCR was performed to examine the mRNA expression of proinflammatory cytokines IL-1β, IL-6, IL-12a, and TNF-α and anti-inflammatory cytokines IL10 and TGFβ in THP1. **(F)** ELISA was performed to assess the protein expression levels of proinflammatory cytokines IL-1β and IL-12p70 and anti-inflammatory cytokine IL-10 in the supernatant of the co-culture. **(G)** qPCR was performed to assess the mRNA expression of iNOS in HMDM. **(H)** qPCR was performed to examine the mRNA expression of proinflammatory cytokines IL-1β, IL-6, IL-12a, and TNF-α and anti-inflammatory cytokines IL-10 and TGF-β in HMDM. Ctrl: uninfected group; *H. pylori* inf: DMSO-pretreated and *H. pylori*-infected group; *H. pylori* inf. + DAPT: DAPT-pretreated and *H. pylori*-infected group. HMDM, human monocyte-derived macrophages. β-actin was used as reference. Data are presented as the mean ± SD of three independent experiments. *p < 0.05, **p < 0.01 and ***p < 0.001, N.S. represents no significant difference.

A growing body of evidence has proven that macrophages polarize to classically activated macrophages (M1) with the production of iNOS and inflammatory cytokines during *H. pylori* infection (Mosser and Edwards, 2008; Martinez et al., 2009; Mege et al., 2011; Martinez and Gordon, 2014). To determine whether Notch signaling participates in this process, RAW264.7 cells were pretreated with DAPT for 24 h and co-cultured with *H. pylori* at an MOI of 50 for 12 h. mRNA expression of iNOS was downregulated, and the release of NO, the product of iNOS, decreased in RAW264.7 compared with the control (Figures 1A,B). Additionally, mRNA expression of pro-inflammatory cytokines IL1β, IL6, and TNFα were significantly lower in DAPT-pretreated RAW264.7 than in untreated cells. However, the mRNA expression of IL12a, one of the units of IL12p70, and IFNγ was lower in DAPT-pretreated RAW264.7 with no significant difference (Figure 1C). Furthermore, the protein expression levels of pro-inflammatory cytokines IL1β and IL6 also significantly decreased, whereas those of IL12p70, TNFα, and IFNγ decreased but insignificantly in DAPT-pretreated RAW264.7 (Figure 1D). However, DAPT-pretreated RAW264.7 did not show a change in the expression of anti-inflammatory cytokines IL-10 and TGFβ (Figures 1C,D).

Considering these results in RAW264.7, we try to replicate the experiments in human macrophages. THP1 and HMDM were used to determine whether Notch signaling is involved in human macrophages during co-culturing with *H. pylori*. The results showed that the expression of pro-inflammatory cytokines THP1, IL1β, IL6, IL12p70, and IFNγ induced by *H. pylori* were significantly reduced in DAPT-pretreated THP1 (Figure 1E). Among these, the protein expression level of inflammatory cytokines IL1β and IL12p70 was significantly reduced in DAPT-pretreated THP1 (Figure 1F). Similarly, the gene expression of the iNOS and pro-inflammatory cytokines (IL1β, IL6, IL12p70, and TNFα) induced by *H. pylori* was significantly reduced in DAPT-pretreated HMDM (Figures 1G,H).

Collectively, these results indicated that Notch signaling is involved in the production of *H. pylori*-induced iNOS and pro-inflammatory cytokines in macrophages.

### Inhibition of Notch Signaling Attenuates Bactericidal Ability of Macrophages to *Helicobacter pylori via* Phagocytosis

**FIGURE 2:**
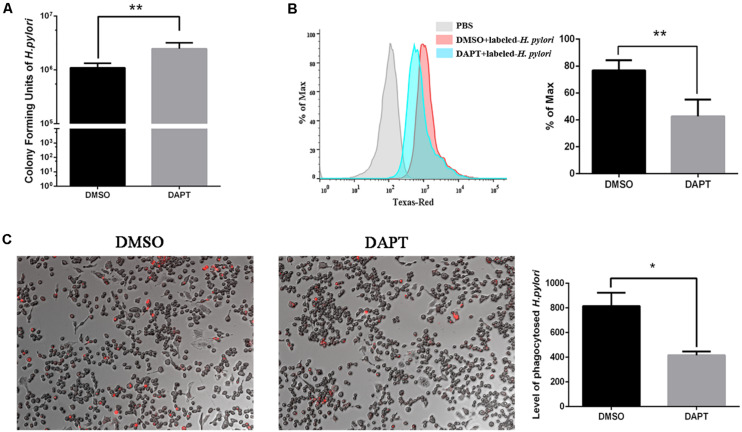
Inhibition of Notch diminishes the bactericidal activity of macrophages to *H. pylori* via phagocytosis. RAW264.7 was pretreated with 40 μM DAPT for 12 h and then were co-cultured with Texas Red-labeled *H. pylori* (MOI = 50) for 3 h. DMSO (0.08%) was used as control for DAPT. PBS was used as control for *H. pylori* infection. **(A)** *H. pylori* in the supernatant was collected and cultured on plates for 7 days to calculate colony-forming units (CFUs) of *H. pylori*. **(B)** The level of Texas Red-labeled *H. pylori* in RAW264.7 cells was determined by flow cytometry. The gray, red, and blue regions represent the background, DMSO-treated RAW264.7 cells, and DAPT-treated RAW264.7 cells, respectively. Representative histograms of Texas Red staining within RAW264.7 cells were assessed by FlowJo_V10 software. PBS group was to distinguish the fluorescence background. **(C)** The Texas Red-labeled *H. pylori* in RAW264.7 cells were observed under a fluorescence microscope. Images were captured using a fluorescence microscope (200×). The red dots represented the Texas Red-labeled *H. pylori*. The total fluorescence intensity of Texas-Red was analyzed by ImageJ (Version 5.12a) software. The histogram reflected the mean fluorescence intensity (MFI). MFI = (the total fluorescence intensity of Texas Red)/(the number of cells). Data are presented as the mean ± SD of three independent experiments. *p < 0.05, **p < 0.01.

To investigate the effect of Notch signaling on the bactericidal ability of macrophages on *H. pylori*, the number of CFU of *H. pylori* was calculated after inhibition of Notch signaling using DAPT. The number of CFU of viable *H. pylori* significantly increased in DAPT-pretreated RAW264.7 compared with DMSO-pretreated cells (Figure 2A), which indicated that the inhibition of Notch signaling attenuates the bactericidal ability of macrophages.

Phagocytosis is associated with the bactericidal activities of macrophages to eradicate *H. pylori* (Fehlings et al., 2012). To investigate the effect of Notch signaling on the phagocytosis of *H. pylori* by macrophages, the Texas Red-labeled *H. pylori* were collected for co-culture with RAW264.7 for 3 h. The flow cytometry and immunofluorescence were performed to assess the phagocytosis of *H. pylori* by RAW264.7. We found that the level of *H. pylori* phagocytosis decreased in DAPT-pretreated macrophages compared with DMSO-pretreated macrophages (Figures 2B,C). These findings suggested that Notch signaling is associated with the bactericidal activities of macrophages *via* phagocytosis of *H. pylori*.

### mRNA and Protein Expression Levels of Jagged1 Are Upregulated in *Helicobacter pylori*-Activated Macrophages

**FIGURE 3:**
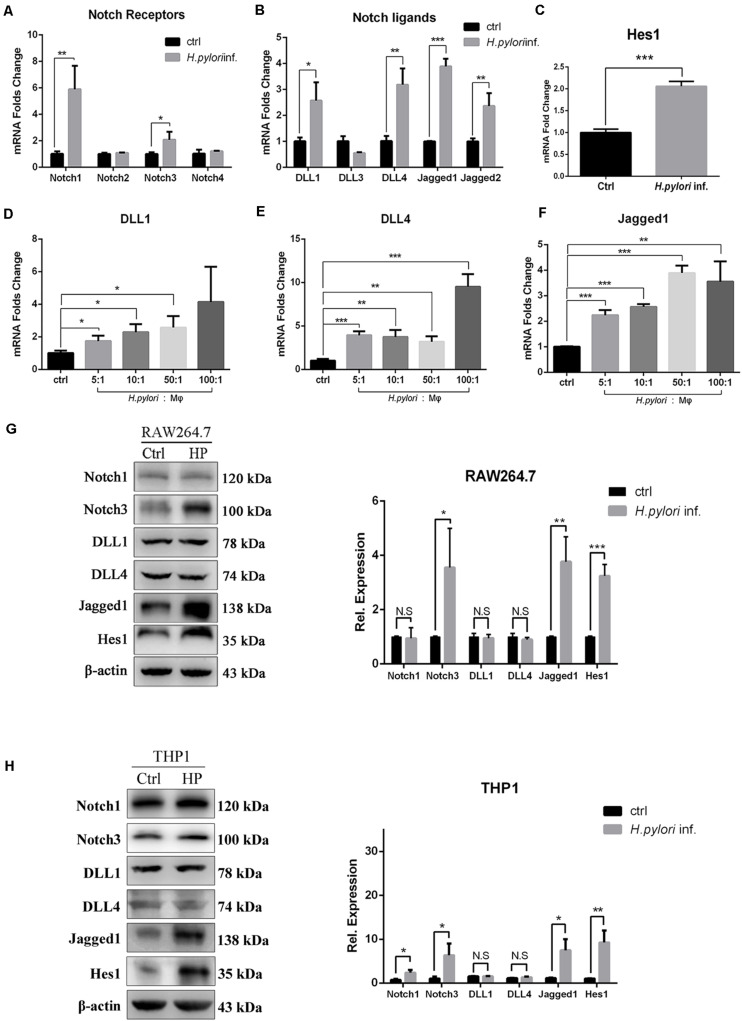
Jagged1 mRNA and protein expression levels are upregulated in *H. pylori*-activated macrophages. RAW264.7 cells were co-cultured with *H. pylori* (MOI = 50) for 12 h. PBS was used as control for *H. pylori* inf. **(A–C)** qPCR was performed to assess the mRNA expression of Notch receptors, Notch ligands, and Notch target gene Hes1 in RAW264.7 cells. PBS was used as control for *H. pylori* infection. **(D–F)** RAW264.7 cells were co-cultured with *H. pylori* using different MOIs (5, 10, 50, and 100) for 12 h. qPCR was performed to examine the mRNA expression of DLL1, DLL4, and Jagged1 in RAW264.7 cells. Macrophages were co-cultured with *H. pylori* (MOI = 50) for 12 h, PBS was used as control for *H. pylori* inf. **(G)** Western blot was performed to assess the protein expression levels of Notch1, Notch3, DLL1, DLL4, Jagged1, and Hes1 in RAW264.7 cells. The expression bands were analyzed using Gel-pro Software, and the integral optical density (IOD) was obtained. The IOD of β-actin was used as reference. **(H)** Western blot was performed to assess the protein expression of Notch1, Notch3, DLL1, DLL4, Jagged1, and Hes1 in THP1. Ctrl: uninfected group; *H. pylori* inf.: *H. pylori*-infected group. β-actin was used as reference. The data are presented as the mean ± SD of three independent experiments. *p < 0.05, **p < 0.01 and ***p < 0.001.

Our results demonstrated that Notch signaling is involved in regulating the activation of macrophages to eradicate *H. pylori*. To explore which Notch receptor or ligand molecules mediate this process, the expression profile of Notch molecules on RAW264.7 co-cultured with *H. pylori* was assessed by using qPCR. The expression levels of Notch1, Notch3, DLL1, DLL4, Jagged1, and Jagged2 were enhanced by 5. 9-, 2. 1-, 2. 6-, 3. 1-, 3. 8-, and 2.5-fold, respectively (Figures 3A,B). In addition, the mRNA expression of Hes1, a Notch signaling downstream target gene, increased by 2.0-fold, indicating that Notch signaling was activated (Figure 3C). For macrophages, scientists pay attention to the role of ligands of Notch signaling, and recent research has shown that DLL1, DLL4, and Jagged1 are involved in macrophage activation to eradicate pathogens (Keewan and Naser, 2020b). Thus, the gene expression of DLL1, DLL4, and Jagged1 was further examined using different MOIs of *H. pylori*. The results showed that the expression of DLL1 and Jagged1 gradually increased in a dose-dependent manner (Figures 3D–F). Furthermore, to investigate whether the increased mRNA expression of Notch ligands coincided with the increased protein expression levels, the protein expression level of Notch1, Notch3, DLL1, DLL4, and Jagged1 was assessed by Western blotting. The results showed that the expression of Jagged1, but not DLL1 and DLL4, and Notch3, not Notch1, were significantly upregulated in macrophages following *H. pylori* stimulation (Figures 3G,H). Furthermore, Western blotting showed that the expression of Hes1 significantly increased, suggesting that Notch signaling was activated. Similar results were obtained for the protein expression levels of Notch1, Notch3, DLL1, DLL4, Jagged1, and Hes1 in *H. pylori*-infected THP1. Taken together, Jagged1 is the only ligand that was upregulated in both mRNA and protein levels in activated macrophages after *H. pylori* infection, which might enhance the inflammatory response to *H. pylori* infection *via* the Notch3-Jagged1 axis.

### Jagged1^+^ CD68^+^ Cells Infiltrate the Gastric Mucosa of *Helicobacter pylori*^+^ Gastritis Patients

**FIGURE 4:**
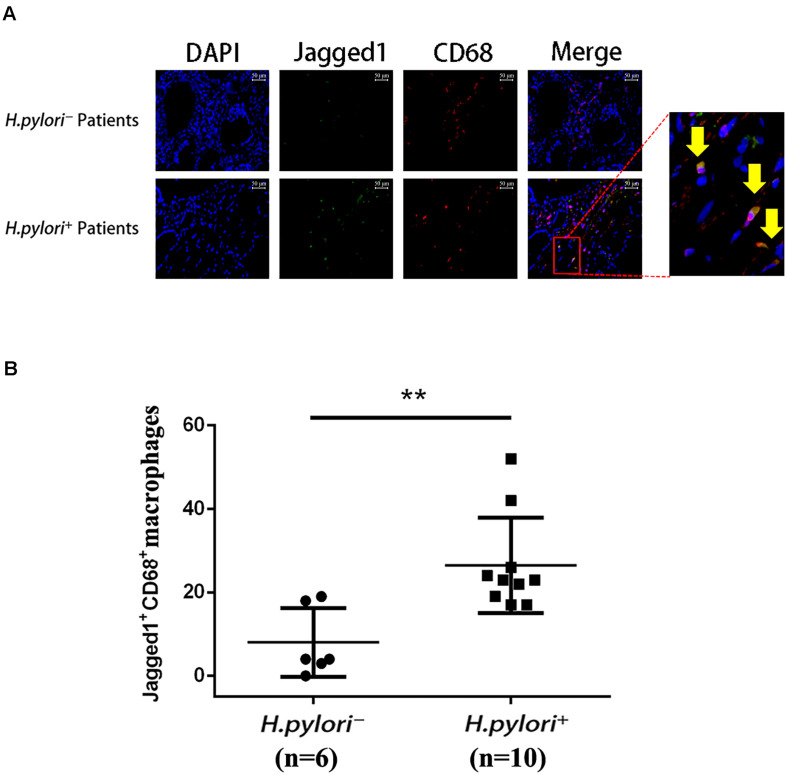
Infiltration of Jagged1^+^ macrophages is higher in the gastric mucosa of *H. pylori*^+^ gastritis patients. **(A)** Gastric mucosa tissues from *H. pylori^–^* and *H. pylori*^+^ gastritis patients were double-immunofluorescent stained with anti-Jagged1 antibody (green) and anti-CD68 antibody (red), and nuclei were stained with DAPI (blue). A merged figure of macrophages is also shown. The yellow arrows indicate Jagged1^+^ CD68^+^ macrophages. Images were observed under a fluorescence microscope (200×). Representative images of the gastric mucosa of *H. pylori^–^* (*n* = 6) or *H. pylori*^+^ (*n* = 10) gastritis patients were shown. The results represent three independent experiments. **(B)** Quantification of the number of CD68^+^ Jagged1^+^ cells in the gastric mucosa of *H. pylori^–^* or *H. pylori*^+^ (*n* = 10) gastritis patients. The data are presented as the mean ± SD of three independent images of each patient. **p < 0.01.

To further investigate the role of Jagged1 in macrophage activation during *H. pylori* infection, we performed immunofluorescence staining of Jagged1 and CD68, a macrophage marker, in the gastric mucosa of *H. pylori-*negative (*H. pylori^–^*) and *H. pylori-*positive (*H. pylori*^+^) gastritis patients. We observed that the infiltration of Jagged1^+^ CD68^+^ macrophages markedly increased in *H. pylori*^+^ subjects compared with *H. pylori^–^* subjects (Figure 4A). Furthermore, we found that the number of the Jagged1^+^ CD68^+^ macrophages was significantly higher in *H. pylori*^+^ subjects compared with *H. pylori^–^* subjects (Figure 4B). These results revealed that *H. pylori* infection is associated with increased expression of Jagged1 in human gastric mucosa macrophages, suggesting that Jagged1 participates in the regulation of macrophage function to eradicate *H. pylori*.

### Jagged1 Overexpression Enhances the Secretion of Pro-inflammatory Mediators and Bactericidal Activities of RAW264.7 Cells

**FIGURE 5:**
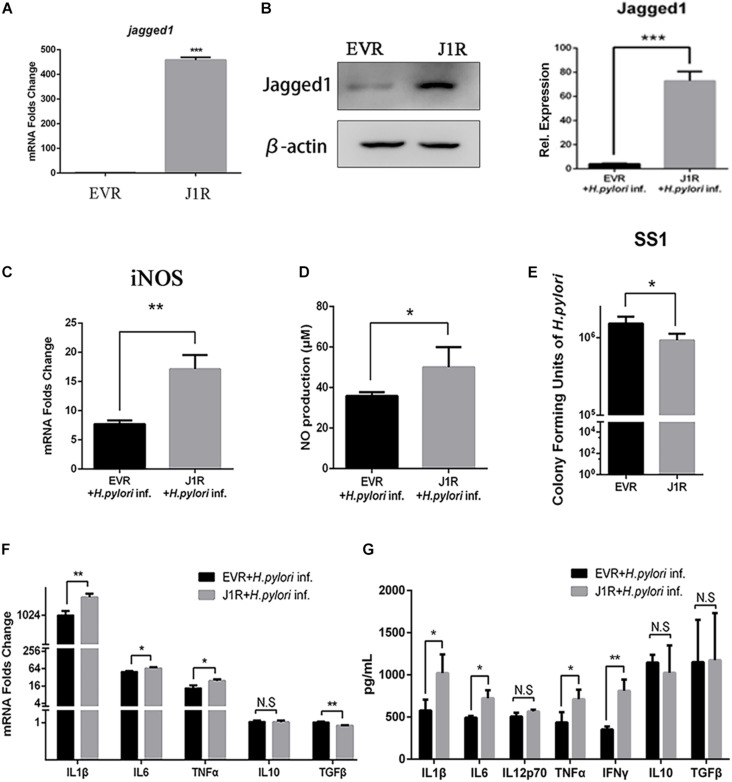
Overexpression of Jagged1 facilitates the secretion of pro-inflammation mediators and bactericidal activity of RAW264.7 cells against *H. pylori*. **(A,B)** The Jagged1-pCMV-Tag4 was transfected into RAW264.7 (J1R) cells, and empty vector transfection was conducted in the control (EVR). Then, the total RNA and total proteins were extracted to verify mRNA and protein expression levels of Jagged1 by qPCR and Western blot analyses. The exposed bands were analyzed by Gel-pro Software by outputting the value of the (IOD). **(C)** qPCR was performed to assess the mRNA expression level of iNOS. **(D)** Griess was performed to assess the production of NO in the supernatant. **(E)** J1R was co-cultured with *H. pylori* (MOI = 50) for 12 h. EVR was used as control for J1R. Then, *H. pylori* in the supernatant was harvested and cultured for 7 days and plated to calculate the number of CFU of *H. pylori*. **(F)** qPCR was performed to assess the mRNA expression levels of pro-inflammatory cytokines IL-1β, IL-6, and TNF-α and anti-inflammatory cytokines IL-10 and TGF-β. **(G)** ELISA was performed to assess the protein expression level of pro-inflammatory cytokines IL-1β, IL-6, IL-12p70, TNF-α, and IFN-γ and anti-inflammatory cytokines IL-10 and TGF-β in the supernatant. β-actin was used as internal control. Data are presented as the mean ± SD of three independent experiments *p < 0.05, **p < 0.01; N.S. represents no significant difference.

To further investigate the role of Jagged1 on macrophage activation during *H. pylori* infection, we screened out stably Jagged1-overexpressing RAW264.7 cells (J1R), which were transfected with the cloning vector (EVR) as control. mRNA and protein expression levels of Jagged1 were both significantly higher in J1R than in EVR (Figures 5A,B), suggesting that J1R was successfully constructed. Then, EVR and J1R were co-cultured with *H. pylori* at an MOI of 50. The mRNA expression of iNOS and the production of NO were significantly upregulated in J1R compared with EVR (Figures 5C,D). The number of CFU of *H. pylori* SS1 decreased in the supernatant of J1R-*H. pylori* co-culture system compared with the control (Figure 5E), which indicated that Jagged1 overexpression in macrophages enhanced the bactericidal activities to *H. pylori*. Additionally, the mRNA expression levels of pro-inflammatory cytokines IL1β, IL6, and TNFα increased in J1R (Figure 5F), and protein secretion of pro-inflammatory cytokines IL1β, IL6, IL12p70, TNFα, and IFNγ was also upregulated in J1R (Figure 5G). However, the expression of anti-inflammatory cytokines IL-10 and TGF-β did not change. Therefore, these results confirmed our hypothesis that Jagged1 participates in regulating macrophages to eradicate *H. pylori*.

### Knockdown of Jagged1 by siRNA Attenuates the Production of NO and Pro-inflammatory Cytokines in RAW264.7 Cells

**FIGURE 6:**
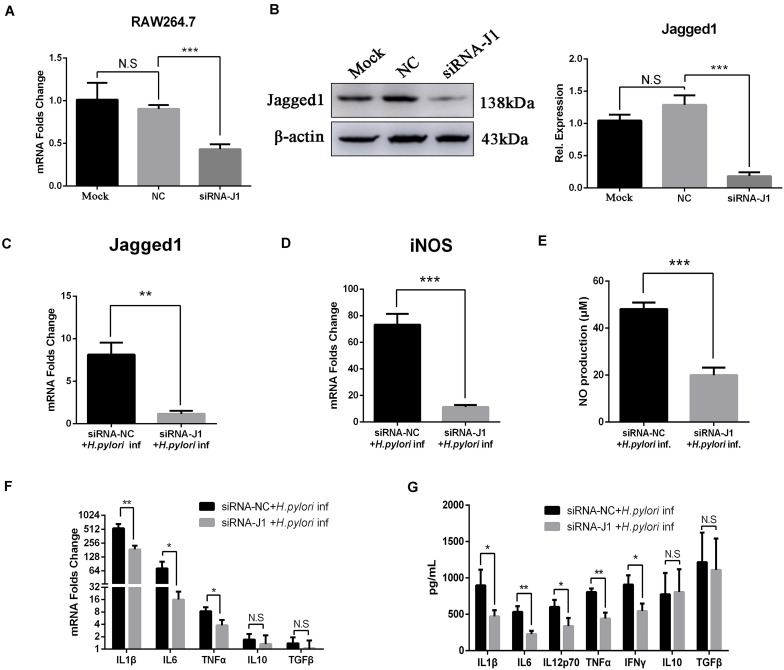
Downregulation of Jagged1 by siRNA decreases the secretion of pro-inflammation mediators in RAW264.7 cells against *H. pylori*. siRNA-J1 (200 nM) was mixed with 2 μl of Lipofectamine 3000. Then, the complex was transfected into RAW264.7 cells for 24 h and then co-cultured with *H. pylori* (MOI = 50) for 12 h. Lipofectamine 3000 and siRNA-NC were used as blank control (Mock) and negative control (NC), respectively. PBS was used as control for *H. pylori* infection. **(A)** Total RNA was extracted after 24 h. qPCR was performed to assess the mRNA expression level of Jagged1. **(B)** Total proteins were extracted after 48 h, and Western blotting was performed to assess the protein expression level of Jagged1. The resulting bands were analyzed by Gel-pro Software, generating the value of the (IOD). **(C)** qPCR was performed to assess the mRNA expression level of Jagged1 in *H. pylori*-stimulated RAW264.7 cells that were treated with siRNA-NC or siRNA-J1 for 48 h. **(D)** qPCR was performed to assess the mRNA expression level of Jagged1 in *H. pylori*-stimulated RAW264.7 cells that were treated with siRNA-NC or siRNA-J1. **(E)** Griess was performed to assess the production of NO in the supernatant. **(F)** qPCR was performed to examine the mRNA expression of proinflammatory cytokines IL-1β, IL-6, and TNF-α and anti-inflammatory cytokines IL-10 and TGF-β. **(G)** ELISA was performed to examine the protein expression levels of pro-inflammatory cytokines IL-1β, IL-6, IL-12p70, TNF-α, and IFN-γ and anti-inflammatory cytokines IL-10 and TGF-β in the supernatant. β-actin was used as reference gene. Data are presented as the mean ± SD of three independent experiments. *p < 0.05, **p < 0.01 and ***p < 0.001; N.S. represents no significant difference.

We previously demonstrated that the overexpression of Jagged1 in macrophages enhances pro-inflammatory cytokine expression and bactericidal activities to eradicate *H. pylori*. Thus, we hypothesized that knockdown of Jagged1 in macrophages would attenuate the responses to *H. pylori*. The siRNA-Jagged1 (siRNA-J1) was utilized to knock down the expression of Jagged1 in RAW264.7. As shown in Figures 6A,B, mRNA and protein expression levels of Jagged1 were significantly lower in siRNA-Jagged1-treated macrophages than the control and NC. Then, siRNA-J1-treated macrophages were co-cultured with *H. pylori* (MOI = 50) for 12 h and their expression for pro-inflammatory mediators was examined. The results showed that *H. pylori*-induced expression of Jagged1 in siRNA-J1-treated macrophages significantly decreased compared with the NC (Figure 6C). Meanwhile, the mRNA expression of iNOS and production of NO were also significantly attenuated in siRNA-J1-treated macrophages compared with the NC (Figures 6D,E). Additionally, the mRNA expression of IL1β, IL6, and TNFα and the secretion of IL1β, IL6, IL12p70, TNFα, and IFNγ were significantly attenuated (Figures 6F,G). Collectively, these data demonstrated that knockdown of Jagged1 significantly attenuated bactericidal activities of macrophages against *H. pylori*, suggesting the key role of Jagged1 in regulating macrophage responses to *H. pylori*.

### Exogenous rJagged1 Triggers Antimicrobial Activities of Macrophages Against *Helicobacter pylori*

**FIGURE 7:**
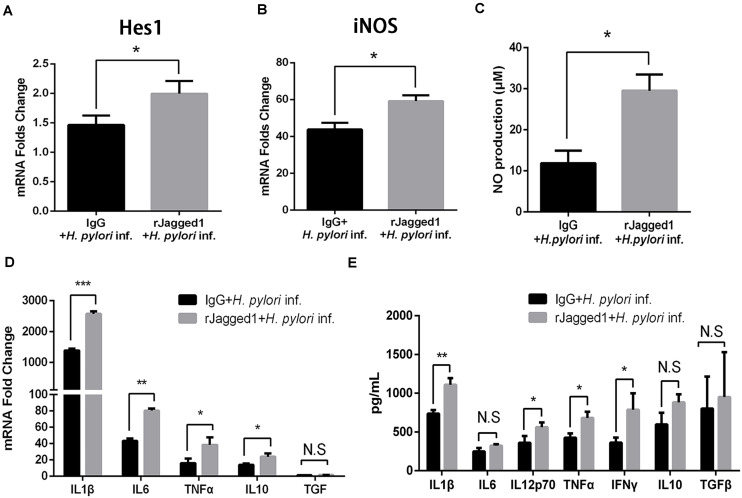
The addition of exogenous rJagged1 enhances the secretion of pro-inflammatory mediators in RAW264.7 cells against *H. pylori* infection. RAW264.7 cells were pretreated with rJagged1 (10 ng/ml) for 24 h and then co-cultured with *H. pylori* (MOI = 50) for 12 h. Human IgG was used as negative control for rJagged1, and PBS was used as control for *H. pylori* infection. Total RNA of each group was extracted. **(A)** qPCR was performed to examine the mRNA expression of Hes1. **(B)** qPCR was performed to examine the mRNA expression of iNOS. **(C)** Griess was performed to test the production of NO in the supernatant. **(D)** qPCR was performed to examine the mRNA expression of pro-inflammatory cytokines IL1β, IL6, and TNFα and anti-inflammatory cytokines IL10 and TGFβ. **(E)** ELISA was performed to examine the protein expression levels of pro-inflammatory cytokines IL1β, IL6, IL12p70, TNFα, and IFNγ and anti-inflammatory cytokines IL10 and TGFβ in the supernatant. β-actin was used as reference gene. Data are presented as the mean ± SD of three independent experiments. *p < 0.05, **p < 0.01 and ***p < 0.001; N.S. represents no significant difference.

For the role of Jagged1, exogenous Jagged1 can also be added in macrophages to explore the function of Jagged1. For example, exogenous rJagged1 activated Notch signaling in macrophages in leprosy and facilitated endothelial cell-driven M1 macrophage differentiation (Kibbie et al., 2016). We next investigated whether rJagged1 promoted macrophage function to eradicate *H. pylori*. RAW264.7 cells were pretreated with rJagged1 (10 ng/ml) for 24 h and then co-cultured with *H. pylori* (MOI = 50) for 12 h. The expression of pro-inflammatory mediators was assessed by qPCR and ELISA. Compared with the control, the mRNA expression of Hes1 was upregulated in rJagged1-pretreated macrophages (Figure 7A), which indicated that rJagged1 reinforced the *H. pylori*-induced activation of Notch signaling. In addition, the mRNA expression of iNOS and the production of NO were significantly upregulated in rJagged1-pretreated macrophages (Figures 7B,C). Furthermore, the mRNA expression of pro-inflammatory cytokines (IL1β, IL6, TNFα, and IL10) and the protein expression levels of pro-inflammatory cytokines (IL1β, IL6, IL12p70, TNFα, and IFNγ) increased in rJagged1-pretreated macrophages (Figures 7D,E). Collectively, these results indicated that rJagged1 enhances the expression of pro-inflammatory mediators of macrophages to eradicate *H. pylori*.

### Anti-Jagged1 Antibody Decreases the Antimicrobial Activities of Macrophages Against *Helicobacter pylori*

**FIGURE 8:**
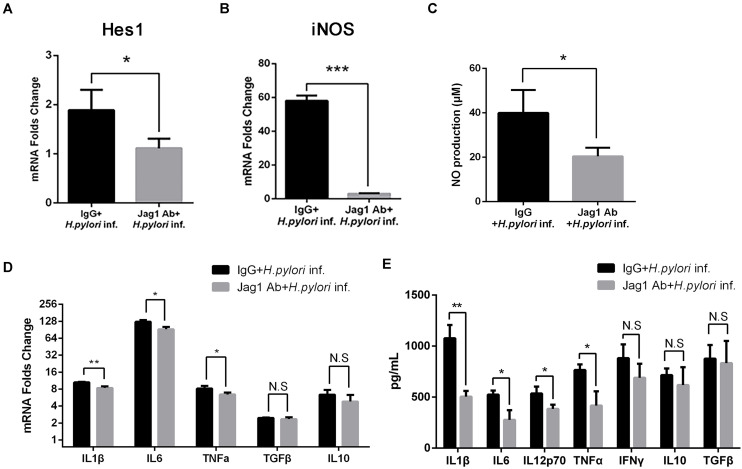
Jagged1 antibody enhances the secretion of pro-inflammatory mediators in RAW264.7 cells against *H. pylori* infection. RAW264.7 cells were pretreated with 3 μg/ml Jagged1 Ab and then co-cultured with *H. pylori* (MOI = 50) for 12 h. Mouse IgG was used as control for anti-Jagged1 Ab. PBS was used as control for *H. pylori*. Total RNA of each group was extracted. **(A)** qPCR was performed to examine the mRNA expression of Hes1. **(B)** qPCR was performed to examine mRNA expression of Jagged1. **(C)** Griess was performed to assess the production of NO in the supernatant. **(D)** qPCR was performed to examine the mRNA expression of proinflammatory cytokines IL1β, IL6, and TNFα and anti-inflammatory cytokines IL10 and TGFβ. **(E)** ELISA was performed to examine the protein level of proinflammatory cytokines IL1β, IL6, IL12p70, TNFα, and IFNγ and anti-inflammatory cytokines IL10 and TGFβ in the supernatant. IgG + *H. pylori* inf: Mouse IgG-pretreated and *H. pylori* infected group; Jag1 Ab + *H. pylori* inf.: anti-Jagged1 Ab-pretreated and *H. pylori*-infected group. β-actin was used as reference gene. Data are presented as the mean ± SD of three independent experiments. *p < 0.05, **p < 0.01 and ***p < 0.001; N.S. represents no significant difference.

To further determine the role of Jagged1 in activated macrophages in *H. pylori* infection, The Jagged1 antibody (Jag1Ab, 3 μg/ml) was added into a co-culture system to block the ligand as an alternative strategy to manipulate Jagged1-initiated Notch signaling. Jag1Ab attenuated the *H. pylori*-induced mRNA expression of Hes1 (Figure 8A), suggesting the inhibition of Notch signaling, which corresponded to the study in which the addition of Jagged1 antibody effectively blocked the axis of the Jagged1-Notch signaling pathway in chronic lymphoid leukemia. The mRNA expression of iNOS and the production of NO both decreased in Jag1Ab-pretreated macrophages (Figures 8B,C). Furthermore, the mRNA expression of pro-inflammatory cytokines (IL1β, IL6, and TNFα) and the protein level of pro-inflammatory cytokines (IL1β, IL6, IL12p70, and TNFα) were attenuated in Jag1Ab-pretreated macrophages, but the expression of anti-inflammatory cytokines (IL10 and TGF-β) did not change (Figures 8D,E). Collectively, these results demonstrated that Jagged1 antibody inhibits Notch signaling and attenuates the *H. pylori*-induced secretion of pro-inflammatory mediators in macrophages.

## Discussion

Macrophages are essential components of the host defense and inflammation to resist the invasion of pathogens (Sica and Mantovani, 2012). In response to various signals from the extracellular milieu, macrophages can be polarized into different populations of activated cells exhibiting different phenotypes and cytokine secretion patterns (Mosser and Edwards, 2008). Classically activated macrophages, also called M1 macrophages, highly express pro-inflammatory cytokines IL-1β, TNF-α, and iNOS to clear the pathogens (Martinez and Gordon, 2014). M2 macrophages are specialized for wound healing with enhanced expression of IL-10 and Arg1 (Martinez and Gordon, 2014). Regulatory macrophages (Mreg) are anti-inflammatory and secrete high levels of IL-10 and TGF-β (Murray and Wynn, 2011). *H. pylori* has been described to induce an enhanced M1-like phenotype (Quiding-Jarbrink et al., 2010; Borlace et al., 2011; Fehlings et al., 2012; Gobert et al., 2014; Moyat and Velin, 2014), representing the expression of iNOS, which would induce NO to combat *H. pylori* infection (Kaparakis et al., 2008; Gobert and Wilson, 2016; Hardbower et al., 2017). M1 macrophages highly express pro-inflammatory cytokines such as IL-1β, IL-6, IL-12p70, TNF-α, and IFN-γ (Lewis et al., 2011; Liao et al., 2016; Wang et al., 2017; Gebremariam et al., 2019). Activation of immune responses by host macrophages upon *H. pylori* infection requires the involvement of a variety of signaling events such as NF-κB and MAPK (Gobert and Wilson, 2017). Recent studies have revealed that Notch signaling is involved in modulating the activation and function of M1 macrophages in resisting invading microbes (Keewan and Naser, 2020a). However, the role of Notch signaling in macrophages against *H. pylori* remains elusive. Our data indicated for the first time that Jagged1, a Notch signaling ligand, plays an important role in M1 macrophage activation and bactericidal activities in combatting *H. pylori* infection.

Jagged1 has been previously found in different types of cells, where it regulates gene expression, thereby modifying the fate or phenotype of cells (Liotta et al., 2008; Mohammad and Guise, 2017; Lee and Long, 2018; Aprile et al., 2020; Jiang et al., 2020). Moreover, there have been some reports showing that Jagged1 expression in macrophages is essential for protection against pathogens. Soluble egg antigen (SEA) of *Schistosoma* could robustly induce the expression of Jagged1 in mouse and human macrophages (Goh et al., 2009). The upregulation of Jagged1 on macrophages regulates the secretion of IL12p40 *via* a potential mechanism to resist *Schistosoma mansoni* infection (Goh et al., 2009; Zheng et al., 2016). The IFNγ-Jagged1 axis instructs macrophage differentiation into M1 macrophages with antimicrobial activity against *M. lepra* (Kibbie et al., 2016). Notch1-Jagged1 signaling regulates specific components of TLR2 responses by NO (Kapoor et al., 2010). In our study, Jagged1 was upregulated in both mRNA and protein levels in macrophages following *H. pylori* stimulation *in vitro*. These results were consistent with the study in which LPS induced Jagged1 mRNA and protein expression in mouse and human macrophages (Goh et al., 2009). Elevated expression of Jagged1 in macrophages is associated with the activation of Notch signaling in hepatic progenitor cells (HPCs) (Li et al., 2018). We thus hypothesized that Jagged1 is a key component of Notch signaling that mediates macrophages against *H. pylori* infection. We further verified that the number of Jagged1^+^ stomach macrophages in *H. pylori*-positive gastritis patients was higher compared with *H. pylori*-negative subjects. In addition, we assessed concomitant induction of Notch signaling downstream gene Hes1. Our findings corresponded with the known ability of Jagged1 to signal *via* the Notch receptor (Lindsell et al., 1995).

During *H. pylori* infection, there are many cells infiltrated in gastric mucosa, including macrophages, neutrophils, and lymphocytes. In our study, to further confirm the role of Jagged1 in regulating macrophage function, we used immunofluorescence staining of Jagged1 and CD68 (a macrophage marker) for gastric mucosa of *H. pylori*-infected patients and healthy controls. The results showed that the infiltration of CD68^+^ Jagged1^+^ macrophages markedly increased in *H. pylori*^+^ subjects compared with *H. pylori^–^* subjects although Jagged1^+^ CD68^+^ macrophages only accounted for a small proportion (Figure 4A). Furthermore, the number of the Jagged1^+^ CD68^+^ macrophages was significantly higher in *H. pylori*^+^ subjects compared with *H. pylori^–^* subjects (Figure 4B), suggesting that Jagged1 participates in the regulation of macrophage function to eradicate *H. pylori*. Of course, there was a large population of other cell types also expressing Jagged1 besides CD68^+^ macrophages, which indicates that other types along with or rather than macrophages may play pivotal roles during *H. pylori* infection. In the future, we will reveal the role of other cell types expressing Jagged1 during *H. pylori* infection.

Moreover, our studies verified that overexpressed Jagged1 on macrophages reinforces the pro-inflammatory function of macrophages during *H. pylori* infection. Downregulated Jagged1 by siRNA alleviated the secretion of pro-inflammatory mediators of macrophages following *H. pylori* infection. To further determine the role of Jagged1 in macrophages to resist *H. pylori* infection, we performed treatment of rJagged1 and Jagged1 neutralizing antibody, as demonstrated in other research (Jung et al., 2007; Kibbie et al., 2016; De Falco et al., 2018). The addition of rJagged1 facilitated the activation of Notch signaling in macrophages stimulated by *H. pylori* and the production of pro-inflammatory mediators, whereas the Jagged1 antibody reversed it.

In addition to the previous reports on the effect of Notch signaling on the differentiation and activation of T/B cells (Algood et al., 2007), this study revealed the possibility that Notch/Jagged1 signaling might participate in the regulation of macrophage activation and polarization during *H. pylori* infection. It is likely that DAPT, a pharmacological inhibitor of Notch signaling, could ameliorate the production of pro-inflammatory mediators and the phagocytosis of macrophages to combat *H. pylori* infection *in vitro*, which coincided with the findings of other research in which DAPT decreased the production of IL-6 and IL-1β in RAW264.7 cells stimulated by LPS, as well as reducing these cytokines *in vivo* (Tsao et al., 2011). In addition, inhibition of Notch signaling in bone marrow macrophages using GSI, an inhibitor of Notch signaling, attenuates LPS/IFN-γ-induced production of IL-6, iNOS, and TNF-α in macrophages (Palaga et al., 2008).

Macrophage differentiation depends on the transcriptional regulator of Notch signaling. Under the regulation of cooperative TLR and Notch signaling, auto-amplification of Notch signaling mediated by the Jagged1-RBP-J axis contributes to macrophage reciprocal regulation in the phenotype (Foldi et al., 2010). Consistent with this result, we observed elevated Jagged1 gene expression in *H. pylori*-activated macrophages. Because M1 macrophages have been reported to be able to activate Notch signaling in co-cultured epithelial cells (Ortiz-Masia et al., 2016) and HPCs (Li et al., 2018), we presumed that macrophage-expressing Jagged1 is a Notch signaling trigger within neighbor cells but is not limited to macrophages.

In addition to the role of Jagged1 in regulating innate immune responses *via* macrophages, evidence has suggested a role for Jagged1 in regulating adaptive T-cell responses. Patients with Alagille syndrome, in which Jagged1 mutations result in a multisystem disorder (Hofmann et al., 2012; Turnpenny and Ellard, 2012), exhibit altered Th1 responses (Le Friec et al., 2012), implicating Jagged1-induced signaling in T-cell differentiation. *In vitro* studies have also shown that Jagged1 expression on keratinocytes promotes dendritic cell maturation, which could influence T-cell responses (Weijzen et al., 2002). Therefore, the expression of Jagged1 by resident cells in tissues can influence both innate and adaptive immune responses. There is accumulating evidence that the interaction of Notch signaling receptors and ligands plays a critical role in macrophages and T-cell communication (Amsen et al., 2004; Rutz et al., 2005). H1N1 influenza virus increases the expression of DLL1 in macrophages, which specifically regulates IFN-γ secretion by CD4^+^ and CD8^+^ T cells both *in vivo* and *in vitro* (Ito et al., 2011). The expression of Notch ligands in macrophages was induced by dengue virus, which is vital for Th1/Th2 differentiation during adaptive immune response (Li et al., 2015). Our research team previously determined that Notch 1 is involved in the differentiation of Th1 cells in *H. pylori* patients (Xie et al., 2020). In our next investigation, we will explore how Jagged1 in macrophages interacts with Notch signaling receptors to affect Th1 cell response during *H. pylori* infection.

In conclusion, Jagged1 is upregulated and associated with macrophage antimicrobial activity against *H. pylori*, thereby suggesting the rationale for new therapeutic approaches that target Jagged1 on macrophages. A novel or additional strategy may involve the combinatorial treatment of Jagged1. The addition of rJagged1 may facilitate macrophage antimicrobial activity to combat *H. pylori* infection. The action of neutralization by Jagged1 may attenuate immunologic injury due to *H. pylori* colonization and alleviate the symptoms of gastritis and ulcer, which facilitate tumor development. Further investigations on its underlying mechanism and *in vivo* investigation are warranted.

There are some limitations in our study. Firstly, all the experiments were conducted *in vitro*. We will complement *in vivo* data to support this finding in *H. pylori*-infected animal model in the future. Secondly, Jagged1^+^ CD68^+^ macrophages only accounted for a small proportion in gastric mucosa of *H. pylori*-infected patients, indicating that there is a large population of other cell types also expressing Jagged1 besides CD68^+^ macrophages. We should also uncover the role of other Jagged1-expressing cell types during *H. pylori* infection in the future. Thirdly, to further illustrate the role of Jagged1 on macrophages against *H. pylori* in human cases, the study needs to involve more experiments with human macrophages to determine it.

## Data Availability Statement

The raw data supporting the conclusions of this article will be made available by the authors, without undue reservation.

## Ethics Statement

The studies involving human participants were reviewed and approved by The Institutional Human Ethics Review Board of Nanfang Hospital, Southern Medical University. The patients/participants provided their written informed consent to participate in this study.

## Author Contributions

JW contributed the majority of the work and wrote the manuscript. CC, ML, XL, JG, TW, SG, and JL participated in the experiment. YN and YL designed and contributed the majority of the work, as well as wrote the manuscript. All authors contributed to the article and approved the submitted version.

## Conflict of Interest

The authors declare that the research was conducted in the absence of any commercial or financial relationships that could be construed as a potential conflict of interest.
