# Supplementary material for: Notch Signaling Ligand Jagged1 Enhances Macrophage-Mediated Response to Helicobacter pylori
Source: Front Microbiol. 2021 Jul 8;12:692832. doi: 10.3389/fmicb.2021.692832 (PMC8297740; doi:10.3389/fmicb.2021.692832)
Supplement: Supplementary file 1 [file Data_Sheet_1.PDF]

Supplementary materials

Table 1 Primer sequences used in qPCR

Species	Target	Sequence (5’ to 3’)
Murine	Notch1	Forward: ACGTAGTCCCACCTGCCTAT
		Reverse: CAGGTGCCCTGATTGTAGCA
Murine	Notch2	Forward: GTTGATCCCCGTCAGTGTGT
		Reverse: CAGGAGGCTGAAGTCGGTTT
Murine	Notch3	Forward: ACTCCTCCTCAGGGAGATGC
		Reverse: GTGGGGTGAAGCCATCAGG
Murine	Notch4	Forward: CCAGAGAGCTTCTGTGTGGA
		Reverse: CAGAAATCCAGGGGCACACT
Murine	DLL1	Forward: ACCAAGTGCCAGTCACAGAG
		Reverse: TCCATCTTACACCTCAGTCGC
Murine	DLL3	Forward: CTCCCGGATGCACTCAACAA
		Reverse: TGGAAGGGGCTGGTATGACA
Murine	DLL4	Forward: CTTTGGCAATGTCTCCACGC
		Reverse: ACTGCCGCTATTCTTGTCCC
Murine	Jagged1	Forward: GGGTCAGTTTGAGCTGGAGA
		Reverse: GTACGTATCACACTCGTCGC
Murine	Jagged2	Forward: GCCTCGTCGTCATTCCCTTT
		Reverse: AGCTCCTCATCTGGAGTGGT
Murine	Hes1	Forward: CCAGCCAGTGTCAACACGA
		Reverse: AATGCCGGGAGCTATCTTTCT
Murine	IL-1β	Forward: TTCAGGCAGGCAGTATCACTC
		Reverse: GAAGGTCCACGGGAAAGACAC
Murine	IL6	Forward: TAGTCCTTCCTACCCCAATTTCC
		Reverse: TTGGTCCTTAGCCACTCCTTC
Murine	IL12a	Forward: CAATCACGCTACCTCCTCTTTT
		Reverse: CAGCAGTGCAGGAATAATGTTTC
Murine	IL12b	Forward: GTCCTCAGAAGCTAACCATCTCC
		Reverse: CCAGAGCCTATGACTCCATGTC
Murine	TNFα	Forward: GGTCACTGTCCCAGCATCTT
		Reverse: CTGTGAAGGGAATGGGTGTT
Murine	IFNγ	Forward: ATGAACGCTACACACTGCATC
		Reverse: CCATCCTTTTGCCAGTTCCTC
Murine	iNOS	Forward: ACCACTCGTACTTGGGATGC
		Reverse: CACCTTGGAGTTCACCCAGT
Murine	IL10	Forward: ATTTCCGATAAGGCTTGGCAA
		Reverse: GCTGGACAACATACTGCTAACC
Murine	TGFβ	Forward: AGTGTGGAGCAACATGTGGAACT
		Reverse: AGCAGCCGGTTACCAAGGTA
Murine	β-actin	Forward: GCAGGAGTACGATGAGTCCG
		Reverse: ACGCAGCTCAGTAACAGTCC
Homo sapiens	IL1β	Forward: ATGATGGCTTATTACAGTGGCAA
		Reverse: GTCGGAGATTCGTAGCTGGA
Homo sapiens	IL6	Forward: CACTGGCAGAAAACAACCTGAA
		Reverse: ACCAGGCAAGTCTCCTCATTGA
Homo sapiens	IL12	Forward: CCTTGCACTTCTGAAGAGATTGA
		Reverse: ACAGGGCCATCATAAAAGAGGT
Homo sapiens	TNFα	Forward: CCTCTCTCTAATCAGCCCTCTG
		Reverse: GAGGACCTGGGAGTAGATGAG
Homo sapiens	IFNγ	Forward: TCGGTAACTGACTTGAATGTCCA
		Reverse: TCGCTTCCCTGTTTTAGCTGC
Homo sapiens	iNOS	Forward: TTCAGTATCACAACCTCAGCAAG
		Reverse: TGGACCTGCAAGTTAAAATCCC
Homo sapiens	IL10	Forward: ACCTGCCTAACATGCTTCGAG
		Reverse: CCAGCTGATCCTTCATTTGAAAG

Homo sapiens	TGFβ	Forward: CTAATGGTGGAAACCCACAACG
		Reverse: TATCGCCAGGAATTGTTGCTG
Homo sapiens	β -actin	Forward: TGGCACCCAGCACAATGAA
		Reverse:CTAAGTCATAGTCCGCCTAGAAGC

Table 2 A list of all antibodies and recombinant proteins used for this study

**Antibodies or recombinant proteins**	**Dilution or concentration**	**Application**	**Source (location)**
Rabbit monoclonal anti-Jagged1	1:1000	WB	Cell signaling, USA Cat. No. 2608
Rabbit polyclonal anti-DLL1	1:1000	WB	Abcam, USA Cat. No. ab10554
Rabbit polyclonal anti-DLL4	1:1000	WB	Abcam, USA Cat. No. ab183532
Anti-β-Actin Mouse Monoclonal Antibody (1C7)	1:5000	WB	Abbkine, USA Cat: No. A01010
Notch1 rabbit polyclonal antibody	1:2000	WB	Proteintech, China Cat: No. 10062-2-AP
Notch3 rabbit polyclonal antibody	1:1000	WB	Proteintech, China Cat: No. 55114-1-AP
Peroxidase Conjugated Goat anti-mouse IgG	1:10000	WB	Fude BioTech, China Cat. No. FDM007
Peroxidase Conjugated Goat anti-Rabbit IgG	1:10000	WB	Fude BioTech, China Cat. No. FDR007
Rabbit polyclonal anti-Jagged1	1:50	IF	Huabio, China Cat. No. R1706-10
Mouse monoclonal anti-CD68	1:100	IF	Huabio, China Cat. No. EM1706-11
Goat anti-mouse IgG, CY3-conjugated	1:300	IF	Servicebio, China Cat. No.GB21301
Goat anti-rabbit IgG, FITC-conjugated	1:300	IF	Servicebio, China Cat. No.GB21303
Recombinant mouse Jagged1 protein (Fc Chimera Active of Human IgG)	10 ng/mL	A	Abcam, USA Cat. No. ab109346
Human IgG	10 ng/mL	A	Dia-an Biotech, China Cat. No. Q6002
Jagged1 neutralizing Antibody	3 μg/ml	Neut	R&D, USA Cat. No. AF1277
Mouse IgG	3 μg/ml	Neut	Proteintech, USA Cat. No. B900620

WB=western blotting, IF=immunofluorescence, A=Activation, Neut=Neutralizing

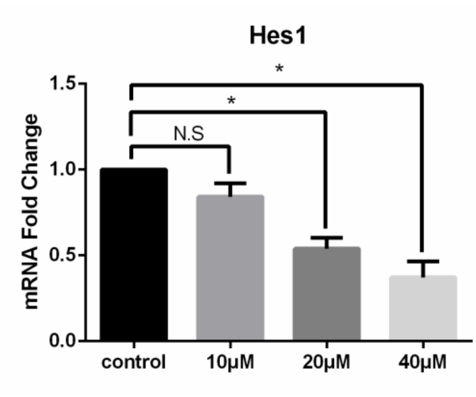

Figure S1: The optimum concentration of DAPT was 40 μM to inhibit Notch signaling. RAW264.7 cells were treated with DAPT (0 μM, 10 μM, 20 μM, 40 μM) for 24 h. The mRNA expression of Hes1, the downstream gene of Notch signaling, was evaluated by qPCR. The histogram represents the mRNA fold change of each DAPT-treated group compared with the control group. β-actin was used as reference gene. Data are presented as the mean ± SD of three independent experiments. * $p<0.05$, N.S represents no significant difference.
